# A Review of the Sentinel Role of *Erinaceus europaeus* in Zoonotic Diseases Across Urban and Rural Environments: A One Health Perspective

**DOI:** 10.3390/vetsci13010029

**Published:** 2025-12-27

**Authors:** Sofia Rosa, Ana C. Silvestre-Ferreira, Felisbina Pereira Queiroga

**Affiliations:** 1 Animal and Veterinary Science Research Centre (CECAV), University of Trás-os-Montes and Alto Douro (UTAD), 5001-801 Vila Real, Portugal; sofiaasrosa99@gmail.com (S.R.);; 2 Department of Veterinary Science, University of Trás-os-Montes and Alto Douro (UTAD), 5001-801 Vila Real, Portugal; 3 Associated Laboratory for Animal and Veterinary Science (AL4AnimalS), 1300-477 Lisboa, Portugal; 4 Center for the Study of Animal Sciences (CECA-ICETA), University of Porto, 4099-002 Porto, Portugal

**Keywords:** hedgehog, zoonotic pathogens, wildlife monitoring, bioindicator, disease ecology, public health, environmental interface

## Abstract

The western-European hedgehog (*Erinaceus europaeus*) is widely distributed across Europe and increasingly interacts with humans and domestic animals due to urban expansion and habitat fragmentation. This review synthesize current knowledge on the zoonotic agents associated with *E. europaeus* and evaluates its potential role as a sentinel species within a One Health framework. Numerous bacteria, viruses, fungi, protozoa, and helminths have been reported in this species, reflecting its ecological adaptability and continuous exposure to diverse environmental sources of infection. The hedgehog’s ability to thrive in both rural and urban settings makes it a valuable bioindicator of environmental health, pathogen circulation and anthropogenic pressures. Understanding the ecological interactions and pathogen dynamics of *E. europaeus* contributes to improving surveillance strategies, supporting integrated approaches to disease prevention and control, and reinforcing the importance of wildlife in One Health initiatives.

## Simple Summary

This review focuses on the western-European hedgehog (*Erinaceus europaeus*), a synanthropic species that frequently lives in proximity to humans and domestic animals. Such coexistence increases the risk of pathogen exposure and transmission, highlighting the hedgehog’s relevance as a sentinel species for zoonotic diseases. Within the One Health framework, this work explores the ecological characteristics of *E. europaeus*, the diversity of pathogens it may harbor, and its importance in understanding the complex interactions between wildlife, humans, and the environment.

## 1. Introduction

### 1.1. Erinaceus europaeus: Ecology, Behavior and Habitat

*Erinaceus europaeus*, commonly known as the western-European hedgehog, belongs to the order *Eulipotyphla*, family *Erinaceidae*, subfamily *Erinaceinae*, and genus *Erinaceus* [1,2]. Four distinct hedgehog species are recognized in Europe: *E. europaeus*, *E. roumanicus*, *E. concolor*, and *Atelerix algirus*. The distribution of *E. europaeus* extends across western and central Europe, including the British Isles, Mediterranean islands, southern Scandinavia, Estonia, and northern Russia, demonstrating a strong ability to adapt to diverse environmental conditions [1,3,4]. This species is considered to exhibit plesiomorphic behavioral traits along with ancestral characteristics in its morphology, physiology, and activity. It is nocturnal and terrestrial, relying mainly on acute hearing and smell [4,5], and is largely solitary, interacting socially only during reproduction or foraging [2,6]. Habitat selection depends on the availability of safe nesting sites, shelter and food. Hedgehogs typically occur in landscapes with shrubs and hedgerows in rural and suburban areas, but can also inhabit forests, grasslands, scrublands and cultivated lands, often preferring humid areas in both Atlantic and Mediterranean regions [7,8]. Their diet consists mainly of insects, snails, slugs, earthworms, and small vertebrates, with beetles serving as the main energy source, although urbanization has increased the consumption of anthropogenic food such as fish, meat, milk and pet food [9,10]. Despite these adaptations, hedgehogs still face natural predation, with badgers representing the primary threat, while dogs, foxes, martens and owls may prey on them opportunistically but usually pose a lower risk [2,11]. To protect themselves, hedgehogs rely on their spines, which cover the body and can be erected in all directions, providing an effective defensive barrier [5].

*E. europaeus* hibernates in response to low temperatures (<8 °C) and short photoperiods, reducing body temperature, respiration, and metabolism between September and May. Before hibernation, individuals build robust nests that function as hibernacula [2,6]. Reproduction begins shortly after hibernation, peaking between May and July and continuing until August. The species is polygamous, with a gestation period of 31–39 days, resulting in the birth of hoglets after about five weeks later [2,12]. Hedgehogs that inhabit urban or suburban areas benefit from structures, food resources and water provided by humans, and population densities tend to be higher in parks and gardens than in forests or large agricultural areas [9].

This species is classified as ‘Least Concern’ by the International Union for Conservation of Nature (IUCN) [13]. However, declines in hedgehog numbers have been reported in recent decades [14]. Road traffic in urban areas remains one of the major causes of mortality for this species [15,16,17], and studies indicate that road traffic can reduce local hedgehog density by nearly 30% [18]. In addition, *E. europaeus* is often exposed to pesticides and rodenticides [19,20], and its survival is further compromised by habitat transformation, fragmentation and the impacts of climate change [21]. Furthermore, studies have confirmed the species’ utility in biomonitoring heavy metal(loid)s, finding elevated concentrations of cadmium (Cd) in kidneys and arsenic (As) linked to geographical provenance, which may have biological effects in hedgehogs, although further research is needed to clarify their impact [22,23,24].

**Figure 1:**
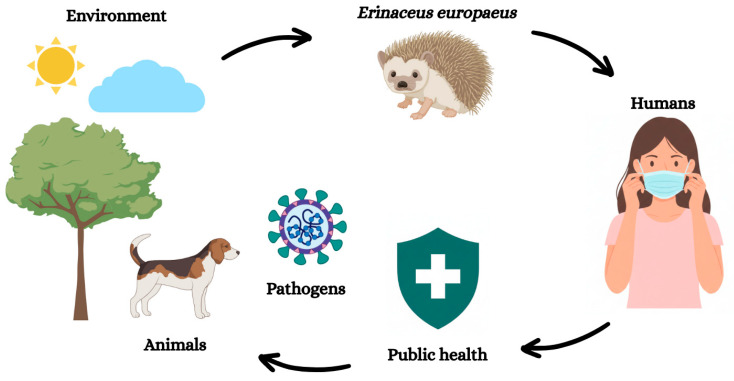
Conceptual representation of the interfaces between the environment, wildlife (including *Erinaceus europaeus*), domestic animals and humans within a One Health framework. The figure illustrates potential pathways of pathogen exposure and circulation rather than depicting complete or pathogen-specific transmission cycles. Interactions are dynamic, multidirectional and influenced by ecological, anthropogenic and environmental factors.

The ecological and feeding habits of hedgehogs, combined with high population densities, synanthropic behavior, and frequent contact with sympatric wild and domestic species, including humans, increase opportunities for pathogen exposure and interspecific interactions. These characteristics help explain their potential role as maintenance, bridge, or terminal hosts in the ecology of several emerging pathogens [25]. As hedgehogs move between natural, rural and urban ecosystems, they are repeatedly exposed to infectious agents, environmental contaminants and ectoparasites, making them increasingly relevant within the broader context of zoonotic disease ecology (Figure 1).

### 1.2. Zoonotic Threats

**Figure 2:**
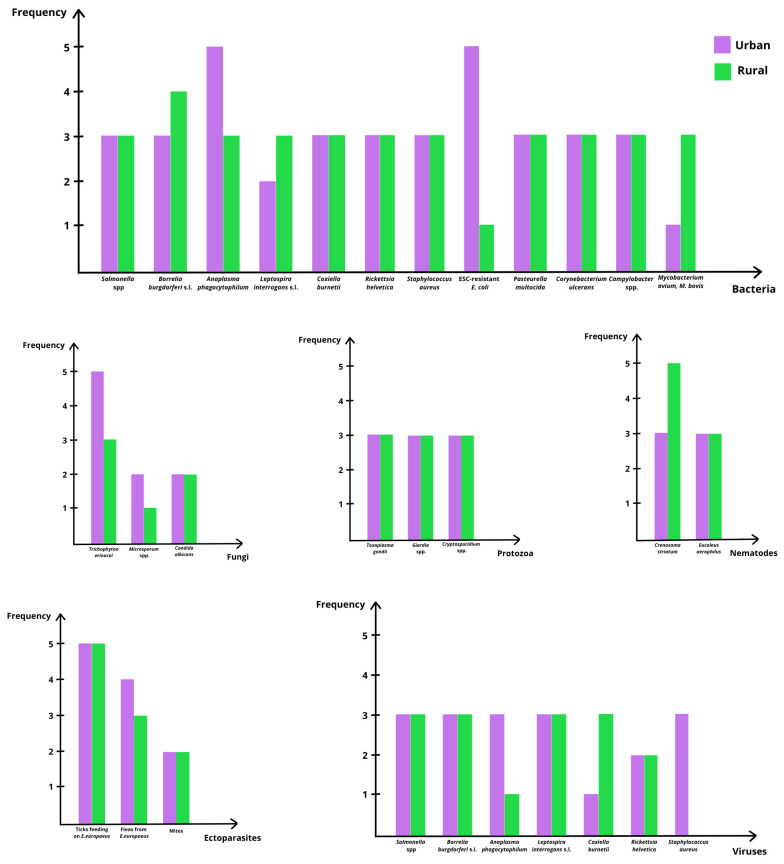
Comparative graphical overview of zoonotic and potentially zoonotic agents detected in *Erinaceus europaeus* across urban and rural environments. The figure provides a visual synthesis of detection frequency patterns for major pathogen and parasite groups using an ordinal classification of occurrence. Each panel represents a specific group of agents and allows qualitative comparison between habitats. This graphical representation is intended to highlight general trends in presence and circulation, rather than quantitative prevalence, acknowledging the heterogeneity of study designs, sampling strategies and diagnostic methods across the available literature. Frequency was classified using an ordinal scale, where 1 = rare or not reported; 2 = occasional; 3 = yes or present; 4 = frequent; and 5 = common/very common.

*Erinaceus europaeus* is increasingly recognized as a reservoir host for a wide range of zoonotic and potentially zoonotic agents, including bacteria, parasites, fungi and viruses [26,27,28]. This capacity reflects the species’ ecological flexibility and its synanthropic behavior, which facilitates exposure to infectious agents across natural, rural and urban habitats [27,28]. As hedgehogs forage in areas used by domestic animals and humans, they encounter contaminated soil, water and vegetation, as well as invertebrate hosts acting as vectors of several pathogens [26,27]. Movement between private gardens, agricultural landscapes and suburban green spaces further increases contact with environmental sources of infection, while human handling during rescue and rehabilitation provides additional opportunities for pathogen exchange [27]. The wide range of microorganisms identified in *E. europaeus* is summarized in Table 1, while a visual comparison of their distribution across urban and rural habitats is presented in Figure 2. Table 1 further details transmission routes and zoonotic relevance.

Parasites are common in *E. europaeus*, although most species have limited direct zoonotic significance. Endoparasites such as *Crenosoma striatum* and *Eucoleus aerophilus* are frequently reported throughout Europe and serve as indicators of environmental contamination and pathogen circulation among wildlife, domestic animals and humans [2,21,27,29]. Protozoa, including *Cryptosporidium* spp., *Giardia* spp. and *Toxoplasma gondii,* have been identified in both urban and rural hedgehogs, reflecting exposure to contaminated water, soil and, in the case of *T. gondii*, oocysts shed by felids [27]. Ectoparasites, particularly ticks of the family Ixodidae, are of significant One Health relevance because hedgehogs may maintain local tick populations in peri-urban areas and contribute to the circulation of vector-borne bacteria such as *Borrelia burgdorferi* sensu lato and *Anaplasma phagocytophilum* [26,27,30,31,32,33,34]. Fleas and mites are also frequently observed, especially in wild individuals, although their zoonotic relevance is comparatively lower [27].

Bacterial infections represent the most substantial zoonotic risk associated with *E. europaeus*. Salmonellosis, particularly involving *S. Enteritidis* and *S. Typhimurium*, is well documented, and hedgehogs often act as asymptomatic carriers capable of contaminating soil, water and fomites through fecal shedding [10,28,35,36]. Additional zoonotic bacteria identified in the species include *Leptospira interrogans* sensu lato, *Coxiella burnetii*, *Rickettsia helvetica*, *Campylobacter* spp. and *Mycobacterium* spp., all of which can be transmitted through direct contact, environmental exposure, or arthropod vectors [10,27,28,37]. The detection of extended-spectrum cephalosporin-resistant *Escherichia coli* in urban hedgehogs is particularly notable, as it reflects exposure to anthropogenic sources of antimicrobial resistance (AMR) and underscores the species’ value as a sentinel for environmental AMR dissemination [30,38]. Other bacterial agents, such as *Pasteurella multocida* and *Corynebacterium ulcerans* have been isolated from hedgehogs, although their zoonotic impact is generally lower and typically associated with direct handling or bite wounds [10].

Hedgehogs also harbor several viruses with varying degrees of known or suspected zoonotic relevance. Erinaceus-associated betacoronaviruses (EriCoVs) have been repeatedly detected in European populations, although their zoonotic potential remains uncertain [25,27,30]. Paramyxoviruses, including Belerina-related strains, have been identified in both wild and captive hedgehogs [25,39], and their ecological and zoonotic significance requires further investigation. Arboviruses such as Tahyna virus have been detected in hedgehogs, and hibernating individuals may function as overwintering reservoirs capable of maintaining viral infectivity during cold seasons [37]. Tick-borne encephalitis virus has also been reported, with hedgehogs demonstrating antibody levels suggestive of repeated exposure [27]. Although rabies virus infection is rare, isolated cases have been confirmed [27,37]. Fungal infections, particularly those caused by *Trichophyton erinacei*, constitute a well-established zoonotic concern [36,38,40,41]. Other fungi, including *Microsporum* spp. and *Candida albicans*, have been documented but have lower zoonotic relevance [42].

Overall, the diversity of zoonotic and potentially zoonotic agents carried by *E. europaeus* highlights its ecological importance at the interface between wildlife, domestic animals, vectors and humans. Although the direct risk to the general public remains relatively low, the species functions as a valuable sentinel for environmental contamination, vector-borne pathogen circulation and antimicrobial resistance within a One Health framework [27,30]. Continued surveillance is therefore essential for improving pathogen detection, assessing health risks and guiding public health and wildlife management strategies.

**Table 1:** Comprehensive overview of pathogens and potentially zoonotic agents documented in *Erinaceus europaeus*.

Group	Agent Detected in *E. europaeus*	Urban	Rural	Transmission Route	Zoonotic Relevance	References
**Bacteria**	*Salmonella* spp. (*S. Enteritidis*, *S. Typhimurium*)	Yes	Yes (low frequency)	Fecal–oral; environmental	High	[10,28,35,36]
	*Borrelia burgdorferi* s.l.	Yes	Yes (frequent)	Tick bites	Moderate to high	[27,30,31]
	*Anaplasma phagocytophilum*	Yes (common)	Yes	Tick bites	Moderate to high	[30,32]
	*Leptospira interrogans* s.l.	Occasional	Present	Urine; environment	Moderate	[27]
	*Coxiella burnetii*	Yes	Yes	Aerosols, environmental	Moderate	[27,28,37]
	*Rickettsia helvetica*	Yes	Yes	Tick bites	Moderate	[27]
	*Staphylococcus aureus* [including MRSA)	Yes	Yes	Contact	Moderate to high	[27,28,37]
	ESC-resistant *Escherichia coli*	Common	Not reported	Fecal contamination	High (AMR)	[30,38]
	*Pasteurella multocida*	Yes	Yes	Bites, scratches	Moderate	[10]
	*Corynebacterium ulcerans*	Yes	Yes	Contact	Moderate	[10]
	*Campylobacter* spp.	Yes	Yes	Fecal–oral	Moderate	[10]
	*Mycobacterium avium*, *M. bovis*	Rare	Present	Aerosols; contact	Moderate	[10]
**Fungi**	*Trichophyton erinacei*	Common	Present	Direct contact	High	[40,41,42,43,44]
	*Microsporum* spp.	Occasional	Rare	Direct contact	Low to moderate	[42]
	*Candida albicans*	Occasional	Occasional	Opportunistic	Low	[42]
**Protozoa**	*Toxoplasma gondii*	Yes	Yes (often higher)	Ingestion of oocysts	Moderate	[27]
	*Giardia* spp.	Yes (including human-hedgehog case)	Yes	Fecal–oral	Moderate	[27]
	*Cryptosporidium* spp.	Yes	Yes	Fecal–oral	Moderate	[26]
**Nematodes**	*Crenosoma striatum*	Yes	Very common	Snails and slugs (intermediate hosts)	Low (not zoonotic)	[2,21,29]
	*Eucoleus aerophilus*	Yes	Yes	Ingestion of eggs	Moderate (rare in humans)	[27]
**Ectoparasites**	Ticks (*Ixodidae*) feeding on *E. europaeus*	Common	Common	Tick bites	High (vector role)	[26,27,33]
	Fleas from *E. europaeus*	Frequent	Present	Contact	Low	[27]
	Mites	Occasional	Occasional	Contact	Low	[27]
**Viruses**	*Erinaceus* coronavirus (EriCoV)	Yes	Yes	Unknown	Unknown (emerging)	[25,27,30]
	Belerina-related paramyxoviruses	Yes	Yes	Unknown	Unknown	[25,39]
	Tahyna virus	Yes	Rare	Mosquito bites	Moderate	[37]
	Tick-borne encephalitis virus	Yes	Yes	Tick bites	Moderate	[27]
	Rabies virus	Rare	Present	Bites	High (rare in hedgehogs)	[27,37]
	Herpesviruses	Occasional	Occasional	Contact	Low	[37]
	SFTSV antibodies detected in *E. europaeus*	Yes	—	Unknown	Unknown	[27]

## 2. One Health Context: Western-European Hedgehogs as Sentinels of Zoonotic Diseases

The *Erinaceus europaeus* occupies a unique ecological position that makes it highly relevant within a One Health framework. Its synanthropic behavior, ability to persist in fragmented and human-dominated landscapes and frequent contact with diverse environmental substrates place it at the interface between wildlife, domestic animals and humans [27,30]. As hedgehogs forage in gardens, parks, agricultural fields and peri-urban green spaces, they are regularly exposed to microorganisms present in soil, water, vegetation and invertebrate hosts, as well as pathogens associated with human activities and companion animals [26,27].

The One Health concept recognizes that the health of humans, animals, and the environment is closely interconnected. Originating from the “One Medicine” vision proposed by veterinarian Calvin Schwabe in the mid-20th century, it emphasizes the need for collaboration among human medicine, veterinary science, ecology, and the social sciences to improve the prevention and control of diseases shared between species [45]. Sentinel species play a crucial role within this framework, as they can signal early signs of infectious threats or environmental disturbances that may ultimately affect humans or other animals [28,46]. Owing to its ecological and feeding habits, high population densities in peri-urban and urban environments, frequent contact with wild and domestic animals, and widespread geographical distribution, *E. europaeus* has substantial potential as a sentinel species. Its biological traits and trophic position support its value in detecting the circulation of multiple pathogens and identifying emerging health risks within a One Health context [25].

Several characteristics reinforce the value of *E. europaeus* as a sentinel species. First, hedgehogs frequently carry ectoparasites, particularly ticks, which serve as vectors for pathogens such as *Borrelia burgdorferi* sensu lato and *Anaplasma phagocytophilum* [30,31,32]. Their ability to sustain tick populations in suburban habitats means that they can act as indicators of both vector abundance and the circulation of emergent tick-borne diseases [26,27,33]. Second, hedgehogs are commonly exposed to environmental contaminants, including pesticides, rodenticides and anthropogenic waste, which may indirectly influence pathogen dynamics and host susceptibility [20,21]. Third, the species’ consistent presence in anthropized landscapes makes it a relevant monitor for environmental AMR. While hedgehogs harbor various bacteria with zoonotic potential, including *Salmonella* spp., *Leptospira interrogans* sensu lato and *Coxiella burnetii*, their key sentinel role in this category is best illustrated by the detection of extended-spectrum cephalosporin-resistant *Escherichia coli* in urban populations, underscoring their relevance as indicators of environmental AMR [30,38].

Hedgehogs also contribute valuable information on fungal, protozoan and viral pathogen circulation. The zoonotic dermatophyte *Trichophyton erinacei* (or *T. mentagrophytes* var. *erinacei*), is frequently detected in *E. europaeus* and represents one of the most relevant pathogens associated with direct human contact. Reported cases in humans include highly inflammatory and pruritic skin eruptions with pustules and thickened lesions, one of which occurred in a pregnant woman who developed extensive dermatitis on her wrist and fingers after handling a hedgehog [41,44]. Another reported case involved kerion tinea barbae, a severe inflammatory form of dermatophytosis affecting the facial region, occurring after direct contact with an infected hedgehog [44].

Evidence of reverse zoonotic transmission has also been reported. A case from New Zealand described Giardia infection in hedgehogs on a farm, traced to a human worker who was actively shedding the parasite, demonstrating that pathogen transmission between humans and hedgehogs can occur in both directions [27].

The epidemiological role of hedgehogs varies by pathogen: hedgehogs act as a reservoir for *Trichophyton erinacei*, facilitating recurrent infections in other hosts, whereas *Giardia* reflects a spill-back reservoir event. Hedgehogs may also act as reservoirs for some variants of *Anaplasma phagocytophilum*, transmitted by ticks. In contrast, agents such as *Coxiella burnetii* may represent incidental reservoirs, where the species is infected but does not sustain endemic cycles [25,27,41,47]. Viral detections, including Erinaceus-associated betacoronaviruses, paramyxoviruses, Tahyna virus and tick-borne encephalitis virus, further expand the relevance of *E. europaeus* in the surveillance of potential emerging diseases, even when the zoonotic significance of several viral agents remains uncertain [25,27,30,37,39]. Although rabies infection in hedgehogs is rare, confirmed cases highlight the importance of maintaining wildlife health surveillance and considering hedgehogs within broader public health monitoring frameworks [27,37].

Importantly, the presence of pathogens alone does not define a sentinel species. According to the National Research Council [46], a sentinel must (i) occupy an interface between humans, domestic animals, and wildlife; (ii) have consistent exposure to environmental sources of infection; (iii) exhibit measurable responses or allow detection of agents reflecting broader trends; and (iv) be suitable for active or passive surveillance. Evidence indicates that hedgehogs serve as sentinels primarily for vector-borne agents, environmental contaminants, and human-origin antimicrobial resistance, while other microorganisms may reflect reservoir or occasional host roles [27,28].

**Figure 3:**
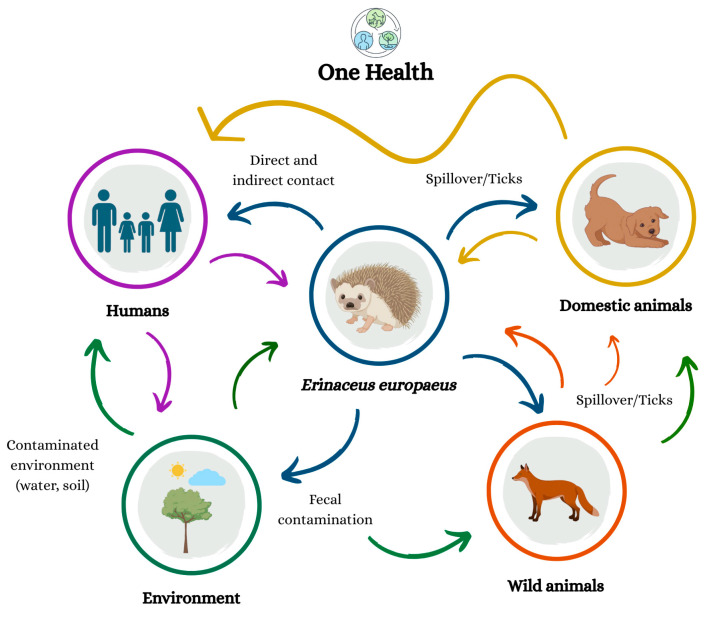
One Health perspective highlighting the ecological interactions between *Erinaceus europaeus*, humans, animals, and the environment, emphasizing the interconnected nature of zoonotic transmission. The arrows indicate the direction of pathogen transmission, where different colors represent the host or environmental compartment from which the agent originates: purple—refers to transmission originating from humans; yellow—refers to transmission originating from domestic animals; orange—refers to transmission originating from wild animals; blue—refers to transmission originating from *Erinaceus europaeus;* and green—refers to transmission originating from the contaminated environment (water, soil). The arrows also describe the main transmission routes, including direct and indirect contact, spillover/ticks, and fecal contamination.

From a One Health perspective, the relevance *of E. europaeus* extends beyond its ability to harbor diverse pathogens. The species functions as an indicator of ecological disturbance, environmental contamination, climate-related changes in parasite communities and shifts in vector distributions, all of which influence human and animal health [21,27,30]. Their frequent interactions with humans, domestic animals, and other wildlife, along with their susceptibility to bacterial, viral, fungal, and protozoan agents, create additional opportunities for pathogen exchange and underscore the importance of biosecurity measures and public education [27]. Integrating data from hedgehogs with information obtained from human and domestic animal cases can enhance the understanding of pathogen circulation across species and help refine targeted public health interventions [28]. Although the direct zoonotic risk to the general public is relatively low, hedgehogs remain a valuable component of integrated surveillance systems, providing early warnings of pathogen emergence, antimicrobial resistance trends and environmental health threats. This surveillance utility is manifested either through the detection of epidemiological shifts in known threats (e.g., rising AMR prevalence or vector expansion) or, in the case of novel zoonotic emergence, through the occurrence of unexplained mass mortality or unusual clinical signs prompting specific diagnostic procedures in rescue centers [27,28,30] (Figure 3).

## 3. Integrated Synthesis and One Health Implications

The *Erinaceus europaeus* is a relevant species within One Health frameworks. Its widespread distribution, synanthropic behavior and ability to persist in fragmented and human-modified habitats create multiple opportunities for contact with pathogens circulating in wildlife, domestic animals and human environments [27,30]. As a result, hedgehogs integrate ecological, environmental and anthropogenic influences, providing meaningful insight into pathogen dynamics at the local and regional scales.

Across Europe, *E. europaeus* consistently demonstrates a high prevalence and diversity of microorganisms, including bacteria, fungi, protozoa, helminths and viruses [10,26,27,28,30]. This diversity reflects not only exposure but also the species’ role as a bridge between ecological compartments. Urban hedgehogs, in particular, tend to carry a greater variety of zoonotic agents, likely due to increased contact with contaminated environments, domestic animals and human-associated waste [30]. The detection of extended-spectrum cephalosporin-resistant *Escherichia coli*, the circulation of *Salmonella* spp. in urban habitats and the presence of tick-borne agents such as *Anaplasma phagocytophilum* and *Borrelia burgdorferi* sensu lato illustrate how hedgehogs can reflect both environmental contamination and vector ecology in areas of intense human activity [28,30,31,32,33,38]. Fungal pathogens such as *Trichophyton erinacei* and the documented case of reverse zoonosis involving Giardia in New Zealand further demonstrate the complexity of interspecies transmission and the bidirectional nature of pathogen flow between humans and wildlife [27,41,44].

From an ecological and epidemiological perspective, these patterns highlight the capacity of *E. europaeus* to act as a sentinel of environmental health. This is exemplified by the detection of heavy metal(loid)s in its tissues, commonly in the kidneys, liver, and spines, where the regional distribution shows a clear pattern of similarity with soil geochemical studies, allowing the identification of local contamination hotspots that may have implications for other animal and human populations [22,48]. Variations in pathogen prevalence between rural and urban habitats, the presence of AMR markers and the detection of viral agents of uncertain zoonotic potential collectively indicate that hedgehogs can reveal early signs of changes in pathogen circulation and environmental disturbance [20,21,27,30]. Because hedgehogs commonly inhabit gardens, parks and peri-urban green areas, they reflect the health risks associated with human-mediated landscape changes, including pollution, habitat fragmentation and altered vector communities.

The implications of these findings extend to both public health and wildlife management. Although the direct zoonotic risk posed by hedgehogs remains relatively low for the general population, close human–hedgehog interactions, particularly during rescue, handling or rehabilitation, can facilitate pathogen exchange and underline the need for appropriate hygiene practices and biosecurity measures [27]. Integrating hedgehog surveillance data with information obtained from humans, domestic animals and vectors can improve understanding of transmission pathways and support targeted public health interventions [28]. More broadly, monitoring hedgehog populations can contribute to the early detection of emerging pathogens, identification of antimicrobial resistance trends and assessment of environmental quality, reinforcing the value of this species within integrated One Health surveillance systems.

Overall, the available evidence demonstrates that *E. europaeus* serves as a sensitive indicator of the interconnected health of humans, animals and ecosystems. Its ecological characteristics and exposure patterns allow it to mirror the impacts of urbanization, environmental contamination and vector dynamics, making hedgehogs a valuable model for understanding and mitigating zoonotic risks in shared environments.

## 4. Knowledge Gaps and Future Research

Despite the growing amount of information on pathogens associated with *E. europaeus*, several important knowledge gaps remain and continue to limit a comprehensive understanding of its zoonotic relevance and its potential role as a One Health sentinel species. Considerable variability in pathogen prevalence across studies and geographic regions has been reported, particularly for agents such as *Anaplasma phagocytophilum* [30,32], *Salmonella* spp. [10,28,35,36], dermatophytes [30,40,42] and hedgehog-associated coronaviruses [25,27,30]. These discrepancies likely reflect differences in sampling strategies, diagnostic sensitivity, local ecological conditions and degrees of urbanization. However, the absence of standardized methodologies makes cross-study comparison difficult and prevents robust epidemiological interpretation. More systematic and harmonized protocols are therefore needed to clarify whether the observed variability represents true ecological differences or methodological divergence.

A further limitation is the scarcity of longitudinal data. Most available studies rely on cross-sectional sampling, commonly using road-killed animals [16,49], individuals admitted to rehabilitation centers [25,49], or opportunistic field collections. While valuable for passive surveillance, these approaches do not provide insight into seasonal patterns, reinfection dynamics, pathogen persistence, or long-term host–pathogen interactions. This gap is particularly relevant for vector-borne pathogens such as *Borrelia burgdorferi* sensu lato and *A. phagocytophilum* [27,30,31,32], whose epidemiology depends on vector activity, climatic factors and host availability. It remains unclear whether hedgehogs act as maintenance hosts, incidental hosts, or bridge hosts in different ecological settings, and this distinction is essential for accurate risk assessment.

Knowledge of cross-species transmission at the human–domestic animal–wildlife interface is also limited. Although documented cases demonstrate that direct zoonotic transmission can occur, such as infection with *Trichophyton erinacei* leading to kerion tinea barbae in humans [41,44] and human outbreaks of *Salmonella Enteritidis* ST183 linked to hedgehogs [35,36], the broader contribution of hedgehogs to community-level transmission is still poorly understood. The detection of antimicrobial-resistant *Escherichia coli* in urban hedgehog populations [30,38] further raises concerns about environmental dissemination of antimicrobial resistance, yet the routes of exposure and the ecological significance of these findings require clarification.

Another major gap concerns the zoonotic potential of viral agents detected in hedgehogs, including paramyxoviruses and Erinaceus-associated coronaviruses [25,27,30,39]. Although their occurrence has been documented in several regions, their host range, mechanisms of transmission and potential for spillover are largely unexplored. More detailed virological and genomic investigations are needed to determine their relevance for human and animal health.

Research on hedgehog immune responses and the effects of environmental stressors on susceptibility to infection is also limited. Handling, captivity, exposure to pesticides and habitat disturbance may weaken immune function [50], but the magnitude of these effects and their impact on pathogen shedding remain unclear [51]. Although reference intervals for hematological and biochemical parameters have been established [26,49,52], further studies are required to evaluate how these parameters vary across regions, seasons and life stages, and to assess their value for population-level health monitoring.

Overall, future research should aim to address methodological inconsistencies, incorporate longitudinal and multicentric designs, investigate cross-species transmission pathways, explore the ecology of antimicrobial resistance, characterize poorly understood viral agents and deepen our understanding of hedgehog physiology and immune function. Strengthening integrated One Health surveillance frameworks that link data from wildlife, domestic animals, vectors, environmental sampling and human health systems will be crucial to clarify the epidemiological role of *E. europaeus* and improve early detection of emerging zoonotic threats in shared environments.

## 5. Conclusions and Future Directions

*Erinaceus europaeus* is an important reservoir and sentinel for a variety of zoonotic pathogens. Its behavior, high population densities, and frequent contact with humans, domestic animals, and wildlife position it as a natural bridge for pathogen circulation across shared environments. Differences observed between urban and rural populations illustrate how habitat characteristics, human activity and environmental pressures shape pathogen prevalence, reinforcing the relevance of this species within a One Health perspective.

Monitoring hedgehogs through passive and active surveillance, including roadkill screening, sampling of clinical cases, and data from wildlife rescue centers, provides valuable information on circulating pathogens and environmental health. When integrated with information from human and domestic animal health systems, these data can support early detection of emerging threats and guide proportionate risk management. Such integrative approaches are particularly important in urban settings, where human–hedgehog interactions are more common and ecological boundaries are increasingly blurred.

Despite growing knowledge, several gaps remain. Some pathogens are poorly studied, and transmission pathways, seasonal trends, and reverse zoonosis are not fully understood. Long-term monitoring and molecular tools are essential to improve detection and understand epidemiology. Comparative analyses of isolates from hedgehogs, humans and domestic animals may further help elucidate interspecies transmission networks and identify critical control points.

Public health interventions, habitat management and educational strategies can reduce zoonotic risks while promoting coexistence with wildlife. Encouraging appropriate hygiene measures during handling, improving environmental quality and strengthening awareness about the ecological role of hedgehogs will contribute to safer and more sustainable human–wildlife interactions. Additionally, assessing the impact of urbanization, climate change and environmental pollutants on hedgehog immunity and susceptibility may reveal broader implications for ecosystem health.

Finally, studying *E. europaeus* as a model for other synanthropic species can provide deeper insight into disease ecology, antimicrobial resistance and the consequences of environmental change. As human-dominated landscapes continue to expand, hedgehogs offer a valuable perspective for understanding pathogen circulation, emerging risks and the interconnected health of animals, humans and ecosystems.

## Data Availability

No new data were created or analyzed in this study.
